# Genome-Wide Detection of Copy Number Variations and Evaluation of Candidate Copy Number Polymorphism Genes Associated With Complex Traits of Pigs

**DOI:** 10.3389/fvets.2022.909039

**Published:** 2022-06-30

**Authors:** Chunlei Zhang, Jing Zhao, Yanli Guo, Qinglei Xu, Mingzheng Liu, Meng Cheng, Xiaohuan Chao, Allan P. Schinckel, Bo Zhou

**Affiliations:** ^1^College of Animal Science and Technology, Nanjing Agricultural University, Nanjing, China; ^2^Department of Animal Sciences, Purdue University, West Lafayette, IN, United States

**Keywords:** evolution, genetic structure analysis, economic traits, livestock, crossbreeding

## Abstract

Copy number variation (CNV) has been considered to be an important source of genetic variation for important phenotypic traits of livestock. In this study, we performed whole-genome CNV detection on Suhuai (SH) (*n* = 23), Chinese Min Zhu (MZ) (*n* = 11), and Large White (LW) (*n* = 12) pigs based on next-generation sequencing data. The copy number variation regions (CNVRs) were annotated and analyzed, and 10,885, 10,836, and 10,917 CNVRs were detected in LW, MZ, and SH pigs, respectively. Some CNVRs have been randomly selected for verification of the variation type by real-time PCR. We found that SH and LW pigs are closely related, while MZ pigs are distantly related to the SH and LW pigs by CNVR-based genetic structure, PCA, V_ST_, and QTL analyses. A total of 14 known genes annotated in CNVRs were unique for LW pigs. Among them, the cyclin T2 (CCNT2) is involved in cell proliferation and the cell cycle. The FA Complementation Group M (FANCM) is involved in defective DNA repair and reproductive cell development. Ten known genes annotated in 47 CNVRs were unique for MZ pigs. The genes included glycerol-3-phosphate acyltransferase 3 (GPAT3) is involved in fat synthesis and is essential to forming the glycerol triphosphate. Glutathione S-transferase mu 4 (GSTM4) gene plays an important role in detoxification. Eleven known genes annotated in 23 CNVRs were unique for SH pigs. Neuroligin 4 X-linked (NLGN4X) and Neuroligin 4 Y-linked (NLGN4Y) are involved with nerve disorders and nerve signal transmission. IgLON family member 5 (IGLON5) is related to autoimmunity and neural activities. The unique characteristics of LW, MZ, and SH pigs are related to these genes with CNV polymorphisms. These findings provide important information for the identification of candidate genes in the molecular breeding of pigs.

## Introduction

Copy number variation (CNV) was discovered in 1936 by Bridges in drosophila (1). The duplication of a segment of the drosophila *Bar* gene caused failure in the formation of normal compound eyes. The definition of CNV is constantly being refined with the additional research. Redon et al. (2) defined CNV as a DNA fragment whose copy number has changed in contrast to the reference genome, and the size from 1 kb to several Mb. According to its structural characteristics, a CNV can be classified as copy number gain or copy number loss. When both copy number gain and loss occur, it is called both type. The CNV mainly affects gene expression through gene dose-effect and gene interruption (3). When the copy number variation region (CNVR) contains dose-sensitive genes, the gene expression level changes with the copy number or the CNV in the coding region influences the gene function and leads to gene disruption and loss of coding ability.

A previous study detected 3,131 CNVRs in Chinese and European pigs. There were 129 and 147 unique CNVRs in Chinese pigs and European pigs, respectively (4). According to the functional enrichment analysis, the genes containing unique CNVRs in Chinese pig breeds are associated with disease resistance and high fertility, while the genes containing unique CNVRs in European pig breeds are closely related to muscle development (4). These results are consistent with the characteristics of Chinese and European pig breeds. A comprehensive CNV study on 98 Xiang pigs and 22 Kele pigs detected 172 CNVRs in 660 annotated genes, which are enriched in sensory, cognitive, reproductive, and ATP synthesis functions (5). These functions are well-matched with the living environment and breed characteristics of Xiang pigs and Kele pigs. In particular, the genes of propagation-related CNVRs have obvious contact with the number of piglets in the Xiang pigs. In addition, studies on the Italian white pig (6), Taihu pig (7), and Bama pig (8) also found a correlation between the breed characteristics and the functions of genes annotated in CNVRs. These studies indicate that the functions of CNVRs are associated with the phenotypes of pigs.

Large White (LW) pigs are well known for their growth and reproductive performance. Min Zhu (MZ) pigs are distributed in northern China and have the characteristics of substantial fat deposition and excellent stress resistance. Suhuai (SH) pigs are crossbred pigs that contain 75 % LW and 25 % Chinese Huai. The Huai and MZ pig breeds originated in north China. The objective of this study is to explore the characteristics of CNV in European LW, Chinese MZ, and crossbred SH pigs at the whole-genome level.

## Materials and Methods

### Samples and Data

Twenty-three SH pigs were selected from the Huaiyin pig farm in Huai'an, Jiangsu Province. A standard phenol/chloroform/isoamyl alcohol protocol was used to extract genomic DNA from pig ear tissue samples. The Illumina Hiseq2000 platform was used for whole-genome sequencing. In addition, the whole-genome sequencing data of MZ pigs (*n* = 11) and LW pigs (*n* = 12) were downloaded from the public database (https://www.ncbi.nlm.nih.gov/) (Supplementary Table 1). The FastQC (https://www.bioinformatics.babraham.ac.uk/projects/fastqc/) was used to analyze the quality of the sequencing data and the parameter was as follows: fastqc -o output -t thread seqfile1..seqfileN. Where “-o” indicates the pathway of the out file, “-t” indicates the number of threads running programs, and “seqfile” indicates the input sequencing data. Then the Cutadapt (https://cutadapt.readthedocs.io/en/stable/) was used for quality filtering and reads trimming. The parameter was as follows: cutadapt -q 10,15 –quality-base = 33 -o output.fastq input.fastq. Where “-q” indicates filtering the quality of the reads, 10 and 15 represent the threshold of the 3' and 5', “–quality-base = 33” indicates the phred33 score system, and “-o” indicates the pathway of the out file. The sequencing data were integrated by MultiQC (v1.11) to meet the requirements of CNV detection (Supplementary Figure 1) (9). The sequences were aligned to the reference genome (Sscrofa 11.1) assembly using the Burrows-Wheeler Aligner (BWA) (v 0.7.17) (10). The overall average sequencing depth reaches 12.89 × , up to 16.22 × , at lowest 9.16 × , and 46 samples' average mapping ratio reached 96.47%.

### CNVR's Definition and Statistics

We used the software CNVcaller to detect CNVs and determine the CNVRs (11). All steps were conducted using the default program. First, build a reference genome database. The reference genome was based on the sliding window of the user's specified size, and the GC, repeat, and gap content of each window on the genome were counted on the genome. The command was as follows: Perl CNVReferenceDB.pl reference.fa -w 800. Where “reference.fa” is the reference genome, “-w” indicates the size of the sliding window. According to the author's suggestion, we selected a window size of 800 bp, and a step of 400 bp to generate the reference genome database. Second, the absolute copy number of each window was calculated. The BAM file (BWA comparison generation) of each sample and the number of reads in each window were analyzed. The high similarity reads (≥97%) were merged, and the low-complexity regions were removed. Based on the GC content, the correct the number of reads in each window after merging was used to calculate the absolute copy number. The command was as follows: bash Individual.Process.sh -b sample.bam -h sample -d link. Where “-b” indicates the BAM file, “-h” indicates the label of the BAM file, and “-d” indicates the link files required for correction. The third step was determination of the CNVR. The boundary of each CNVR was preliminarily determined by comprehensively considering the distribution of absolute copy number, the frequency of variation, and the significant correlation between adjacent windows (primaryCNVR). Then, the adjacent CNVRs whose copy number distribution was significantly related to the population were further merged to obtain the final CNV detection results (mergedCNVR). The command was as follows: bash CNV.Discovery.sh -l list -e exclude_list -f 0.1 -h 1 -r 0.5 -p primaryCNVR -m mergeCNVR. Where “-l” indicates the list of results files after the absolute copy number correction; “-e” indicates the samples in this list are not used for the detection of CNVR. “-f” and “-h” represent the difference between the individual's absolute copy number and the reference absolute copy number in frequency and quantity, which greater than the setting value is considered a candidate CNV window; “-r” indicates the correlation coefficient of the absolute copy number of the adjacent candidate CNV window (no overlap), which greater than the setting value will be merged; and “-p” and “-m” indicate the output files primaryCNVR and mergeCNVR. A genome-wide CNVR map was drawn by RIdeogram (12).

### Genetic Structure Analysis

The CNVRs detected were used to analyze the genetic structure differences among three pig breeds. We performed principal component analysis (PCA) by PLINK (v 1.90) (13). PLINK was used to convert the CNVRs file into bed format. ADMIXTURE (v 1.3.0) was used to execute population genetic structure analysis (14). We first set the ancestral population number K value between 1 and 5, then compute the Cross-Validation Error for each K values. When the Cross-Validation Error value became the least, the K value was the number of ancestors. MEGAX was used for evolutionary tree analysis to evaluate the genetic distance between the populations. By calculating the V_ST_ value (2), we analyze the genetic difference between the two groups.


$$\begin{array}{l} {\text{V}}_{\text{ST}}=\frac{V_{total}-\frac{V_{1}\times N_{1}+V_{2}\times N_{2}}{N_{total}}}{V_{total}} \end{array}$$

Where V_total_ is the total variance in copy number between the two groups, V_1_ and V_2_ are variances in copy number of population 1 and population 2, respectively. N_1_ and N_2_ are the numbers of samples of population 1 and population 2, respectively. N_total_ is the total number of all the samples. We compare the genetic distance between groups by the mean V_ST_ values. All diagrams were drawn by ggplot2 (15, 16).

### CNVR Annotation and Population Differences Comparison

To further study the relationship between CNVRs and the phenotypic characteristics of the population, a Venn diagram was drawn by TBTOOLS (v 1.098661) (17) to observe the differential and common CNVRs. Gene annotation and pathway enrichment were conducted for the population-specific CNVRs using g:Profiler (18) and KOBAS (19), respectively.

### Group-Specific CNVR Overlapped With QTLs

QTL data were downloaded from Pig QTLdb (https://www.animalgenome.org/cgi-bin/QTLdb/SS/index). Bedtools (v 2.15.0) (20) was used to overlap the QTLs with the group-specific CNVRs, and the unique corresponding QTL area was obtained after removing the repeat value. According to the description of QTL traits, the group-specific CNVRs that affect the phenotypes of LW, MZ, and SH pigs were analyzed.

### Validation of Quantitative Real-Time PCR

We randomly selected 4 CNVRs fragments to detect copy number polymorphisms by qPCR and the 2^−Δ*Ct*^ method, ΔCt value = (Ct_target_ – Ct_reference_) (21). Primers used in qPCR were designed by Primer-BLAST (https://www.ncbi.nlm.nih.gov/tools/primer-blast). The highly conserved fragment of the *GCG* in pigs was selected as an internal reference gene (22). Primer sequences for CNVRs and *GCG* are shown in Supplementary Table 2. To ensure that the test samples were comparable to the *GCG*, we first constructed the standard curve of each CNVR after gradient dilution of DNA. Total CNVs were verified on the QuantStudio 5 real-time PCR system (ABI, USA), and PCR amplification conditions were designed according to the manufacturer's description (Vazyme, China). The PCR amplification system was completed in a 20 μL system, including the following ingredients: 10 μL SYBR master Mix, 2 μL DNA (around 5ng), 0.4 μL forward primers, and 0.4 μL reverse primers, and 7.2 μL water. The PCR conditions were as follows: first step 95° C for 30 seconds followed by 40 cycles at 95 ° C for 10 s and 60 ° C for 30 s. The CNV type detected by the above PCR method was the same as those detected by the CNVcaller. Where CNVR-9017 was the gain type in LW pigs, but the normal type in SH pigs. The CNVR-1169, CNVR-9126, and CNVR-1771 were expressed in two pig breeds as the gain type. In addition, we used the Integrative Genomics Viewer (IGV) (23) to visualize the genome of the samples, and its results were the same as qPCR (Supplementary Figure 2). Each CNVR fragment has 4 biological repetitions in both LW and SH pigs, and all samples were performed in triplicate.

## Results

### CNVR Detection and Statistics

**Figure 1:**
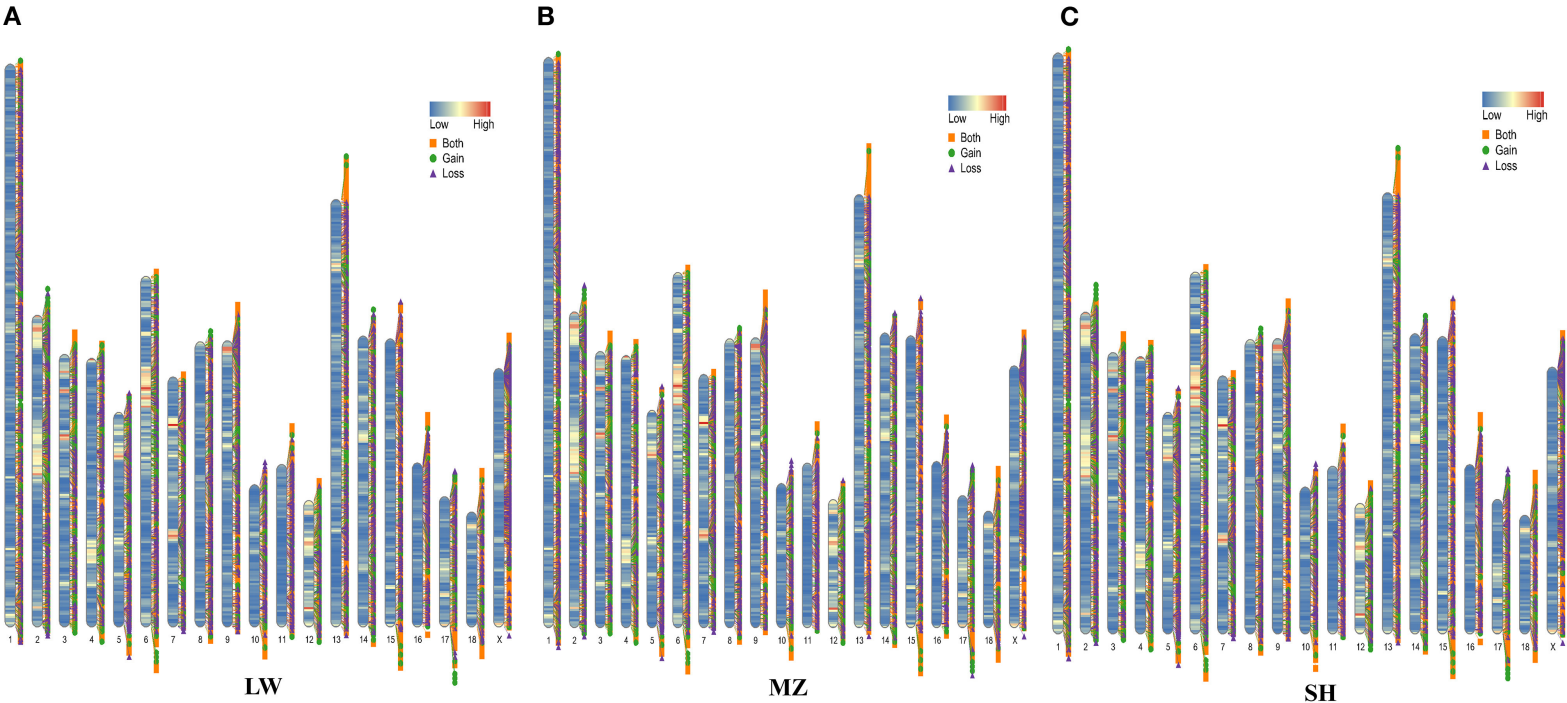
The genome distribution of CNVRs and variation types of LW **(A)**, MZ **(B)**, and SH **(C)** pigs. The legend of “Low” to “High” indicates the gene density on the pig chromosomes. The yellow square represents the both type, the green circle represents the gain type, and the purple triangle represents the loss type.

A total of 11,173 CNVRs were detected in 46 pigs (Supplementary Table 3). There were 10,917, 10,885, and 10,836 CNVRs detected in SH, LW, and MZ pigs, respectively. The coverage area of these CNVRs in the three populations is more than 43 million bp, which accounts for about 1.8% of the whole genome (Sscrofa 11.1) (Supplementary Table 4). In all samples, there were 3,457, 2390, and 5,326 cases of copy number loss, copy number gain, and both type, respectively (Figure 1). The length of CNVRs ranges from 1.6 to 560 kb, but 61.23% of CNVRs are 1.6 to 3 kb, and only 0.75% CNVRs are more than 30 kb (Supplementary Figure 3). Moreover, a total of 8,247 CNVRs were detected in <5 pigs, and 4,134 CNVRs were found in the unique individual (Supplementary Figure 4).

### Analysis of Population Clustering

**Figure 2:**
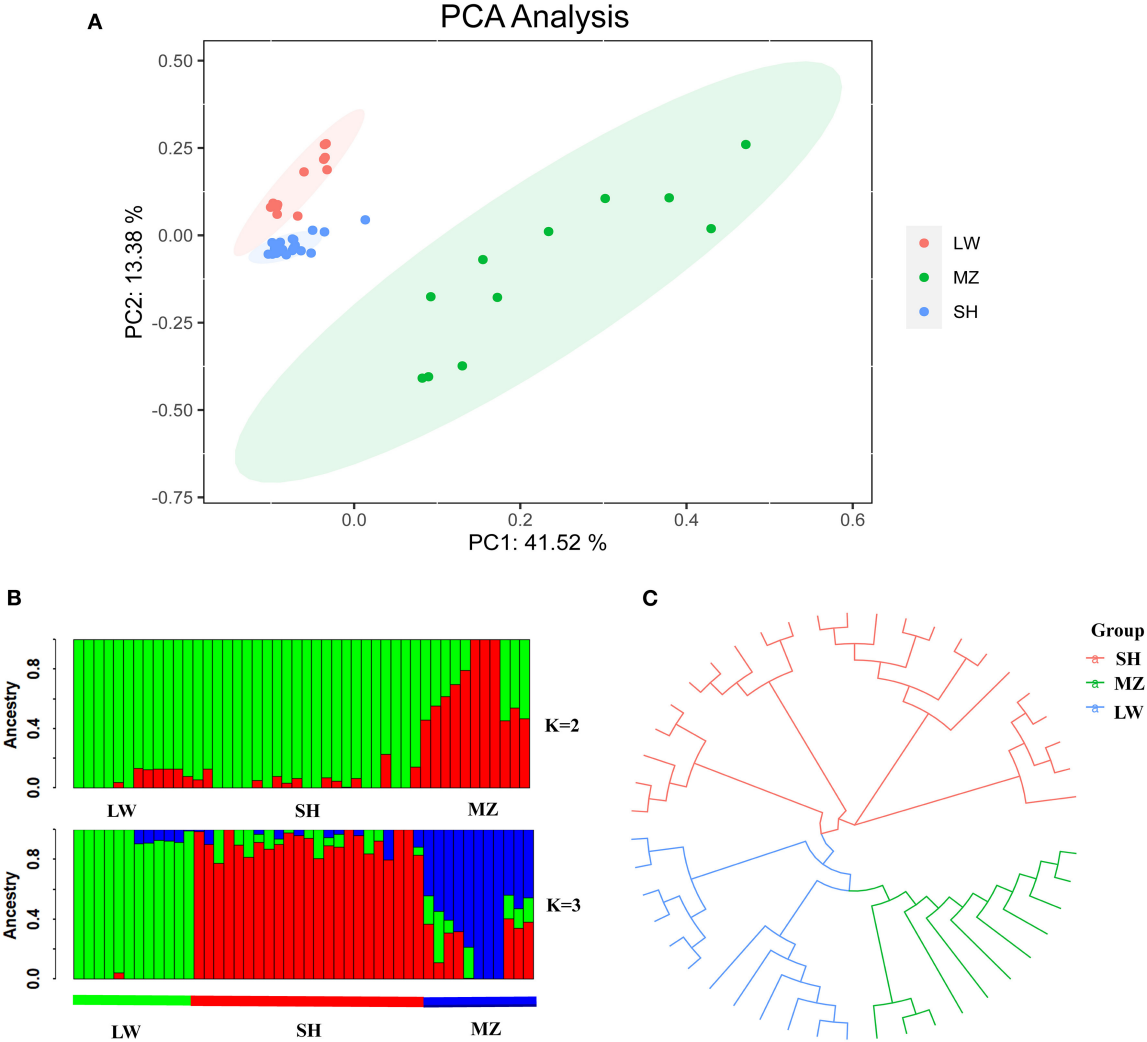
**(A)** PCA plot of LW, MZ, and SH pigs. Red, green, and blue represent LW, MZ, and SH pigs, respectively. **(B)** Diagram of genetic structure analysis, the K value represents the number of the hypothetical ancestor. When the ancestral population number K = 2, there are obvious differences between LW and MZ pigs, while the information of SH pigs is covered by LW pigs, green and red represent LW and MZ pigs, respectively. When K = 3, the three pig breeds are well separated. Green, red, and blue represent LW, SH, and MZ pigs, respectively. **(C)** Evolutionary tree diagram of LW, SH, and MZ pigs. The location of SH pigs is closer to the root, moreover, the genetic distance between SH and LW pigs is less than SH and MZ pigs.

A PCA graph was developed with all the samples having been divided into three groups: SH, MZ, and LW pig breeds (Figure 2A). The LW and SH pigs are closer in the PCA diagram, and the individuals are arrayed tight. The MZ pigs are far from them, and the individuals are scattered.

### Genetic Structure Analysis

**Figure 3:**
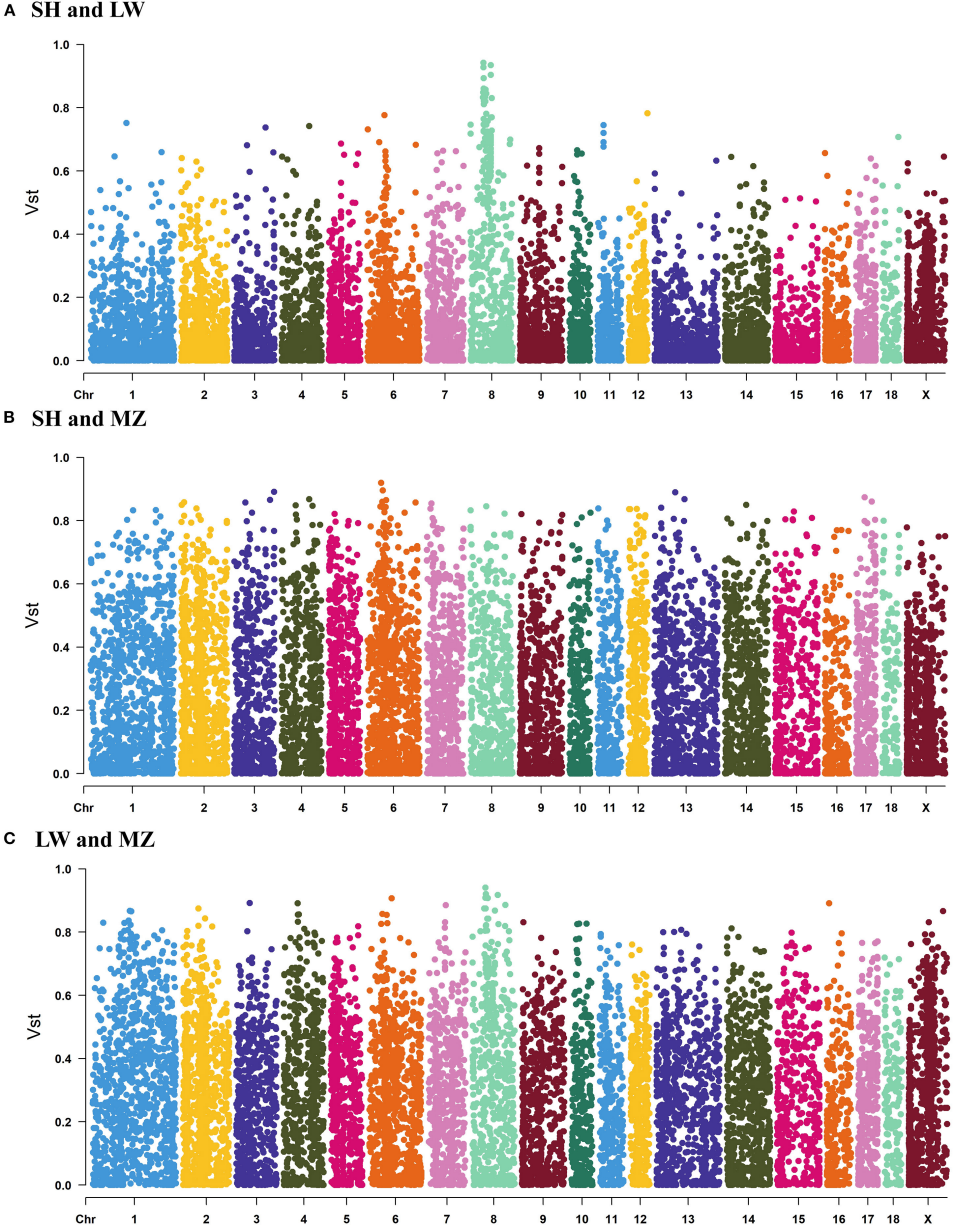
The V_ST_ values of all the copy number variation regions (CNVRs) in SH and LW **(A)** pigs, SH and MZ **(B)** pigs, LW and MZ **(C)** pigs. The average VST value of SH and LW pigs is just 0.111; but the average VST values are 0.234 and 0.265 in SH and MZ pigs and LW and MZ pigs, respectively.

When the ancestral population number K = 2, there are obvious differences between LW and MZ pigs, while the information of SH pigs is covered by that of LW pigs. When K = 3, the Cross-Validation Error is the smallest (Supplementary Table 5), and the three pig breeds are well separated (Figure 2B). The result of phylogenetic tree analysis is similar to that of PCA. Since the genetic background of the SH pig is complicated (containing 75 % Large White and 25 % Chinese Huai), the position of the SH pigs is close to the root of the tree, and the distance to LW pigs is closer than MZ pigs (Figure 2C). The average V_ST_ value of SH and LW pigs is just 0.111; but the average V_ST_ values are 0.234 and 0.265 in SH and MZ pigs and LW and MZ pigs, respectively (Figure 3). The V_ST_ analysis results are the same as the PCA analysis and genetic structural analysis. The genetic distance between SH and LW pigs is smaller than that between LW and MZ pigs.

### Analysis of Shared and Group-Specific CNVR

**Figure 4:**
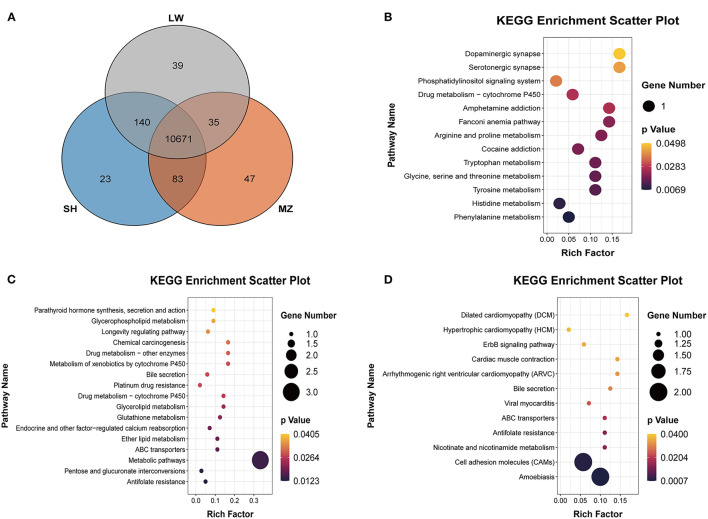
**(A)** Venn diagram of the CNVRs in LW, SH, and MZ pigs. The known genes of LW **(B)**, MZ **(C)**, and SH **(D)** pigs in the group-specific CNVRs were analyzed in the KEGG pathway.

The differences in CNVRs between pig breeds were compared through the Venn diagram (Figure 4A). A total of 10,671 CNVRs are shared among the three pig breeds. There are 23, 47, and 39 group-specific CNVRs in the SH, MZ, and LW pigs, respectively. A total of 140 CNVRs are common in the SH and LW pigs, while only 83 CNVRs are common in the SH and MZ pigs, and 35 CNVRs are common in the LW and MZ pigs.

### Gene Research in Group-Specific CNVR

We noted the genes associated with group-specific CNVRs and discovered 35 known genes (Table 1) and 25 novel genes (Supplementary Table 6). These known genes were analyzed in the KEGG pathway.

**Table 1:** Annotated genes in group-Specific CNVRs of LW, MZ, and SH pigs.

**Population**	**Ensemble_id**	**Gene_name**	**Description**
	ENSSSCG00000032786	ZC3HAV1L	Zinc finger CCCH-type containing, antiviral 1 like
	ENSSSCG00000024999	PPIP5K2	Diphosphoinositol pentakisphosphate kinase 2
	ENSSSCG00000025784	CDH4	Cadherin 4
	ENSSSCG00000015830	UNC5D	Unc-5 netrin receptor D
	ENSSSCG00000005000	FANCM	FA complementation group M
	ENSSSCG00000016141	PLEKHM3	Pleckstrin homology domain containing M3
LW	ENSSSCG00000015379	DNAH11	Dynein axonemal heavy chain 11
	ENSSSCG00000001975	PRKD1	Protein kinase D1
	ENSSSCG00000015697	CCNT2	Cyclin T2
	ENSSSCG00000023215	MAOB	Monoamine oxidase B
	ENSSSCG00000012101	ANOS1	Anosmin 1
	ENSSSCG00000042659	ZSCAN5A	Zinc finger and SCAN domain containing 5A
	ENSSSCG00000042659	ZSCAN5B	Zinc finger and SCAN domain containing 5B
	ENSSSCG00000042659	ZSCAN5C	Zinc finger and SCAN domain containing 5C
	ENSSSCG00000009347	KL	Klotho
	ENSSSCG00000044340	EEA1	Early endosome antigen 1
	ENSSSCG00000027349	TBC1D14	TBC1 domain family member 14
	ENSSSCG00000009233	GPAT3	Glycerol-3-phosphate acyltransferase 3
	ENSSSCG00000007727	AUTS2	Activator of transcription and developmental regulator AUTS2
MZ	ENSSSCG00000030262	GDPD1	Glycerophosphodiester phosphodiesterase domain containing 1
	ENSSSCG00000038036	TTLL11	Tubulin tyrosine ligase like 11
	ENSSSCG00000037808	GSTM4	Glutathione S-transferase mu 4
	ENSSSCG00000021846	EFHC2	EF-hand domain containing 2
	ENSSSCG00000009497	ABCC4	ATP binding cassette subfamily C member 4
	ENSSSCG00000011040	CACNB2	Calcium voltage-gated channel auxiliary subunit beta 2
	ENSSSCG00000015435	NAMPT	Nicotinamide phosphoribosyltransferase
	ENSSSCG00000023934	KCNIP4	Potassium voltage-gated channel interacting protein 4
	ENSSSCG00000009215	ABCG2	ATP binding cassette subfamily G member 2 (Junior blood group)
	ENSSSCG00000033643	NLGN4X	Neuroligin 4 X-linked
SH	ENSSSCG00000033643	NLGN4Y	Neuroligin 4 Y-linked
	ENSSSCG00000033560	SERPINB3	Serpin family B member 3
	ENSSSCG00000033560	SERPINB4	Serpin family B member 4
	ENSSSCG00000011121	CELF2	CUGBP Elav-like family member 2
	ENSSSCG00000003227	IGLON5	IgLON family member 5
	ENSSSCG00000024674	ABL2	ABL proto-oncogene 2, non-receptor tyrosine kinase

A total of 14 known genes were annotated in 39 unique CNVRs in LW pigs. These genes regulate the metabolism of phenylalanine, histidine, and other amino acids based on the KEGG pathway (Figure 4B). The *CCNT2* gene is widely involved in the regulation of cell differentiation and the cell cycle. In fibroblasts of C2C12 cells, the overexpression of *CCNT2* strengthened *MyoD*-dependent transcription and promoted myogenic differentiation (24). A comprehensive study reported that the *CCNT2* gene induced the differentiation of muscle cells with the molecular partner *Pkn* (25), which may play a positive role in the meat production of LW pigs. The *FANCM* gene is involved in defective DNA repair and reproductive cell development (26). Previous studies found that the *FANCM* gene was associated with Non-obstructive Azoospermia and ovarian deficiency, which led to male/female infertility (27, 28). It may be related to the reproductive performance of the LW pigs. LW pigs are commonly mated to other maternal lines to produce crossbred commercial sows.

We annotated 10 known genes in 47 unique CNVRs in MZ pigs. These genes are enriched in “Antifolate Resistance,” “Metabolic Pathways,” and “Glycerolipid Metabolism” based on the KEGG pathway (Figure 4C). A previous study reported that the *GPAT3* gene plays an important role in lipid metabolism, which causes rapid growth and exquisite meat quality in Yunling cattle (29). The knockout of the *GPAT3* gene altered energy balance in diet-induced obesity in mice, indicating that the *GPAT3* gene plays a role in regulating energy and lipid homeostasis (30). It may be related to the fat deposition capacity of MZ pigs. The *GSTM4* and *TBC1D14* genes are considered to participate in detoxification and autophagy (31, 32). These genes are related to “Glutathione Metabolism,” “Platinum Drug Resistance,” and “Metabolism of Xenobiotics by Cytochrome P450” detoxification and resistance gene pathways.

We have annotated 11 known genes in the 23 unique CNVRs in SH pigs. These genes are enriched in resistance and ATP-related pathways (Figure 4D). Interestingly, some genes are associated with neurodevelopment. The *NLGN4X* and *NLGN4Y* genes are located on the X and Y chromosomes, respectively. Neurogenesis, neuron differentiation, and muscle development are increasingly disturbed in neuron stem cells with NLGN4X knockdown, including DLG4 and NLGN3 postsynaptic genes also have decreased expression (33). The *IGLON5* gene participates in regulating sleep and other neural activities and is also related to autoimmunity (34).

### Group-Specific CNVRs Overlapped With QTLs

**Figure 5:**
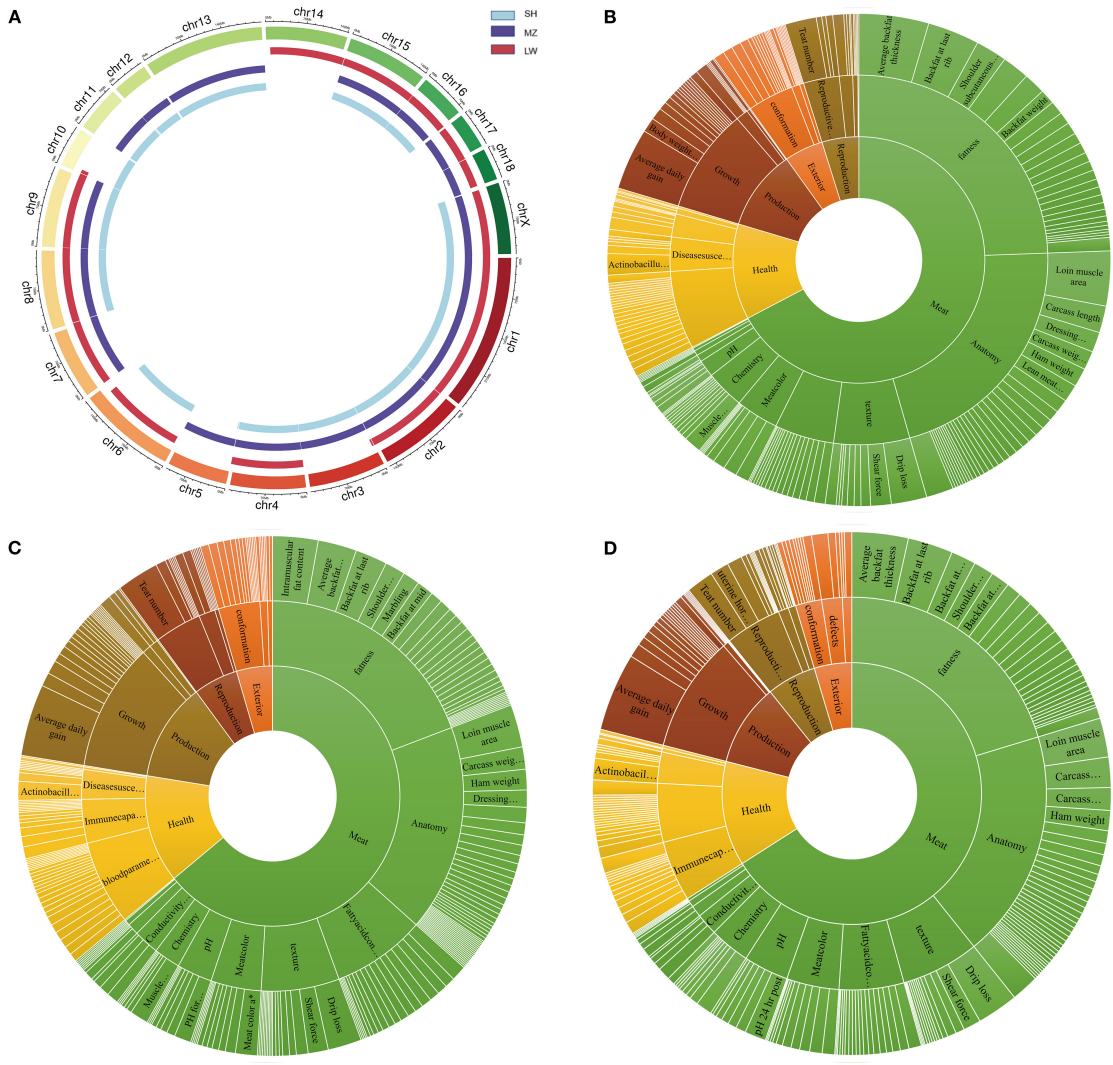
**(A)** The genome distribution of the group-specific QTLs in SH, MZ, and LW pigs. **(B–D)** are the group-specific QTLs in SH, LW, and MZ pigs.

The group-specific CNVRs of LW, SH, and MZ pigs were mapped in the QTLs of the pigs. There are 1,139, 938, and 1,283 QTLs in the SH, LW, and MZ pigs, respectively. A Venn diagram shows that 248 QTLs overlap between the LW and SH pigs, 237 QTLs overlap between the SH and MZ pigs, and 178 QTLs overlap between the MZ and LW pigs. There are 285, 545, and 700 group-specific QTLs in the SH, LW, and MZ pigs, respectively (Supplementary Figure 5). A circus diagram was used to show the location of these unique QTLs (Figure 5A). The effects of QTLs on traits are divided into three levels, “Trait Categories,” “Trait Type,” and “Trait.” The difference in the meat and disease resistance traits of LW, MZ, and SH pigs is more distinct (35) (Figures 5B–D). So QTLs for meat and health trait categories were analyzed.

In the anatomy type of the meat category, the trait cases of “muscle area and muscle fiber” and “fat to meat ratio and fat-cut percentage” are different in LW, MZ, and SH pigs. The number of muscle-related QTLs is 12.2 times that of fat-related QTLs in LW pigs (61/5). And this ratio is only 3.6 times and 7 times in MZ and SH pigs (55/15, 35/5). Interestingly, the “EnzyMeactivity” QTLs are unique to the MZ pigs. The number of total “NADPH-generation enzyme activity” and “NADP-malate dehydrogenase activity” is 12, which is related to the oxidation reaction in the organism, particularly fatty acids generation (36). In the trait category of health, the number of “Immune capacity” is huge difference among LW, MZ, and SH pigs, with a total of 24 traits, and 64 QTLs related to immune capacity in the MZ pigs, but only 6 traits, 23 QTLs, and 14 traits, 34 QTLs are in LW and SH pigs, respectively.

## Discussion

The role of CNV's is an increasingly discussed academic topic, and previous studies on CNV have been conducted in humans, cattle, sheep, and other species (37–40). CNVs could destroy the normal expression of genes and ultimately cause phenotypic changes mainly through dosage effects, interruption, and position effects of gene deletion and duplication (41–43). As a type of essential variation in the genome, CNV polymorphisms play key roles in species evolution, environmental adaptation, disease resistance, and disease susceptibility (44–46). However, numerous past studies have concentrated on CNV on the chromosomal DNA with little attention given to CNV of non-chromosomal DNA. Mitochondrial DNA (mtDNA) passes through maternal inheritance, which has been confirmed to be related to many traits, including respiratory and cardiovascular disease (47). As a component of ribosomes, rRNA easily becomes a substrate of homologous recombination resulting in CNV due to its repetitive sequence structure (48).

In our present study, we noticed that LW pigs have excellent meat production. Several genes containing the unique CNVRs are involved in the regulation of cell proliferation and cell cycle regulation in LW pigs. These genes have extensive participation in muscle growth and development. We also obtained the same results in the QTL analysis.

Among these genes, the *CCNT2* gene is related to cell differentiation and cell cycle, especially regulating the differentiation of muscle cells (24). Many studies have focused on the combined analysis of microRNA (miRNA) and *CCNT2*. Previous research reported that miR-15a, miR-155-5p, and miR-188-5p inhibit muscle differentiation and skeletal muscle development *via* target binding *CCNT2* (49–51). Due to their great reproductive performance, in the modern pig breeding systems, LW pigs are used to produce crossbred female parents. Among these genes, *FANCM* is involved in DNA damage repair, and the mutation causes deaths of spermatogenic cells at all levels and stagnation of round spermatids, which causes male reproductive disorders, including sperm deformities, decreased motility, and decreased numbers (27). These results are interesting because these genes may be related to the reproductive performance of LW pigs.

We found *GPAT3* related to adipogenesis in unique CNVRs in MZ pigs. The promoter polymorphisms of the *GPAT3* were associated with intramuscular fat content in Laiwu pigs, and the knockout of *GPAT3* was related to insulin resistance and fatty liver in a mouse model of severe congenital generalized lipodystrophy (30, 52). The *GPAT3* accelerated the fat production capacity of MZ pigs. Understandably, the habitat of the MZ pigs is in northern China, where winter temperatures reach minus 40 degrees Celsius. Sufficient fat keeps them resistant to the cold and stores energy. Similarly, MZ pigs have good disease resistance and detoxification capabilities. *GSTM4* is a member of the glutathione sulfur transferase family and plays a key role in the detoxification of insecticides and other exogenous substances. In abamectin-resistant *tetranychus urticae*, the activity of GSTs was significantly increased (53). The QTLs mapped to the group-specific CNVRs in MZ pigs are related to fat and immunity. The genes mentioned above provide favorable conditions for the survival of MZ pigs in cold regions.

The SH pig is crossbred of Chinese and European pigs. The CNV polymorphisms of some genes were unique in SH pigs. *SERPINB3* is a homologous substance to chicken ovalbumin protein (OVA) in humans. It takes part in apoptosis and autoimmune diseases and is related to the prognosis (54). The *NAMPT* is primarily involved in redox reactions, and the signals it transmits act during various stages of cell physiology, including cell cycle and proliferation (55). It is a participant and regulator of many diseases. The results were within our expectations, including genes related to immunity and cell proliferation. What surprised us was that some genes are related to neuroprotection and neurological disorders. *NLGN4X* and *NLGN4Y*, as marker molecules of human autism, are considered to play an important role in the etiology of autism, the formation of synapses, and the transmission of information. Autism can lead to stereotypic behavior and communication difficulties in humans and is related to developmental mental disorders (56, 57). In addition, the massive accumulation of *IGLON5* antibodies has been proven to damage the cytoskeleton of hippocampal neurons, which can lead to the occurrence of autoimmune diseases and neurodegeneration (34, 58). These findings were interesting as SH pigs are more docile and more easily domesticated than LW pigs. The neurological foundation of these behavioral differences is still unknown.

By analyzing the genetic structure of LW, MZ, and SH pigs, we found that SH and LW pigs are closely related, while MZ pigs are distantly related to pigs of the other two breeds. It indicates that LW and SH pigs have more genetic exchanges than MZ pigs, which have the same trend in PCA, evolutionary tree, V_ST_, and the group-special CNVRs and QTLs analyses. Based on the results of genetic structural analysis, we found that the lineage of SH pigs came from LW pigs, and MZ pigs have a smaller genetic distance from SH pigs than LW pigs. This may be because the MZ pig have genetic exchanges with the LW pig of widespread reproduction, and the habitats of MZ and SH pigs are similar in geographical location, climate, and altitude, which have the same environmental driving forces and adaptability that make them produce the same CNV (59). Understandably, the main source of CNV was inherited from ancestors, followed by adaptation to environmental changes and other reasons that led to random mutations (60, 61).

## Conclusion

In summary, we have performed genome-wide CNV detection on LW, MZ, and SH pigs to explore the relationship between CNVs and phenotypic characteristics of pig breeds. The functions of genes containing unique CNVRs are related to the phenotypic traits of pig breeds. From this, we have identified some candidate genes. These CNV polymorphisms provide a theoretical basis for the understanding of the relationship between phenotype and CNVs.

## Data Availability Statement

The datasets presented in this study can be found in online repositories. The names of the repository/repositories and accession number(s) can be found in the article/Supplementary Material.

## Ethics Statement

The animal study was reviewed and approved by Experimental Animal Welfare and Ethics Committee of Nanjing Agricultural University, Nanjing, China.

## Author Contributions

BZ came up with the idea and revised the manuscript. CZ wrote the manuscript and performed the experiments. JZ, YG, and QX collected the samples and isolated the genomic DNA. ML, MC, and XC analyzed the data. AS and BZ reviewed and edited the manuscript. All authors have read and agreed to the published version of the manuscript.

## Funding

This work was supported by the National Natural Science Foundation of China (NO. 32172786) and the JBGS Project of Breeding Industry Revitalization in Jiangsu Province [JBGS(2021)101].

## Conflict of Interest

The authors declare that the research was conducted in the absence of any commercial or financial relationships that could be construed as a potential conflict of interest.

## Publisher's Note

All claims expressed in this article are solely those of the authors and do not necessarily represent those of their affiliated organizations, or those of the publisher, the editors and the reviewers. Any product that may be evaluated in this article, or claim that may be made by its manufacturer, is not guaranteed or endorsed by the publisher.

## Supplementary Material

Supplementary Figure 1 All samples are suitable for CNV detection.

Supplementary Figure 2 Genomic visualization of CNVR-9017, CNVR-1169, CNVR-9126, and CNVR-1771 in LW and SH pigs.

Supplementary Figure 3 The length-frequency distribution of CNVRs. The majority of CNVRs are concentrated in 1.6-3 kb, accounting for 61.23% of the total, with only 0.75% exceeding 30 kb.

Supplementary Figure 4 The variable frequency distribution of CNVRs. A total of 8,247 CNVRs were found in <5 individuals, and 4,134 CNVRs were found in a unique individual.

Supplementary Figure 5 A Venn diagram shows 285, 545, and 700 group-specific QTLs in the SH, LW, and MZ pigs, respectively.

Supplementary Table 1 The whole-genome sequencing data of MZ, LW, and SH pigs.

Supplementary Table 2 The standard curve and primers for qPCR, and the verification results of the CNV type.

Supplementary Table 3 A total of CNVRs were detected in 46 pigs and variations of types.

Supplementary Table 4 The distribution of CNVRs on pig chromosomes of the pig.

Supplementary Table 5 The Cross-Validation Error under the ancestral population number K value ranges from 1 to 5.

Supplementary Table 6 Novel genes identified in LW, MZ, and SH pigs.
