# Supplementary figures and images for: Genome-Wide Detection of Copy Number Variations and Evaluation of Candidate Copy Number Polymorphism Genes Associated With Complex Traits of Pigs
Source: Front Vet Sci. 2022 Jun 30;9:909039. doi: 10.3389/fvets.2022.909039 (PMC9280686; doi:10.3389/fvets.2022.909039)

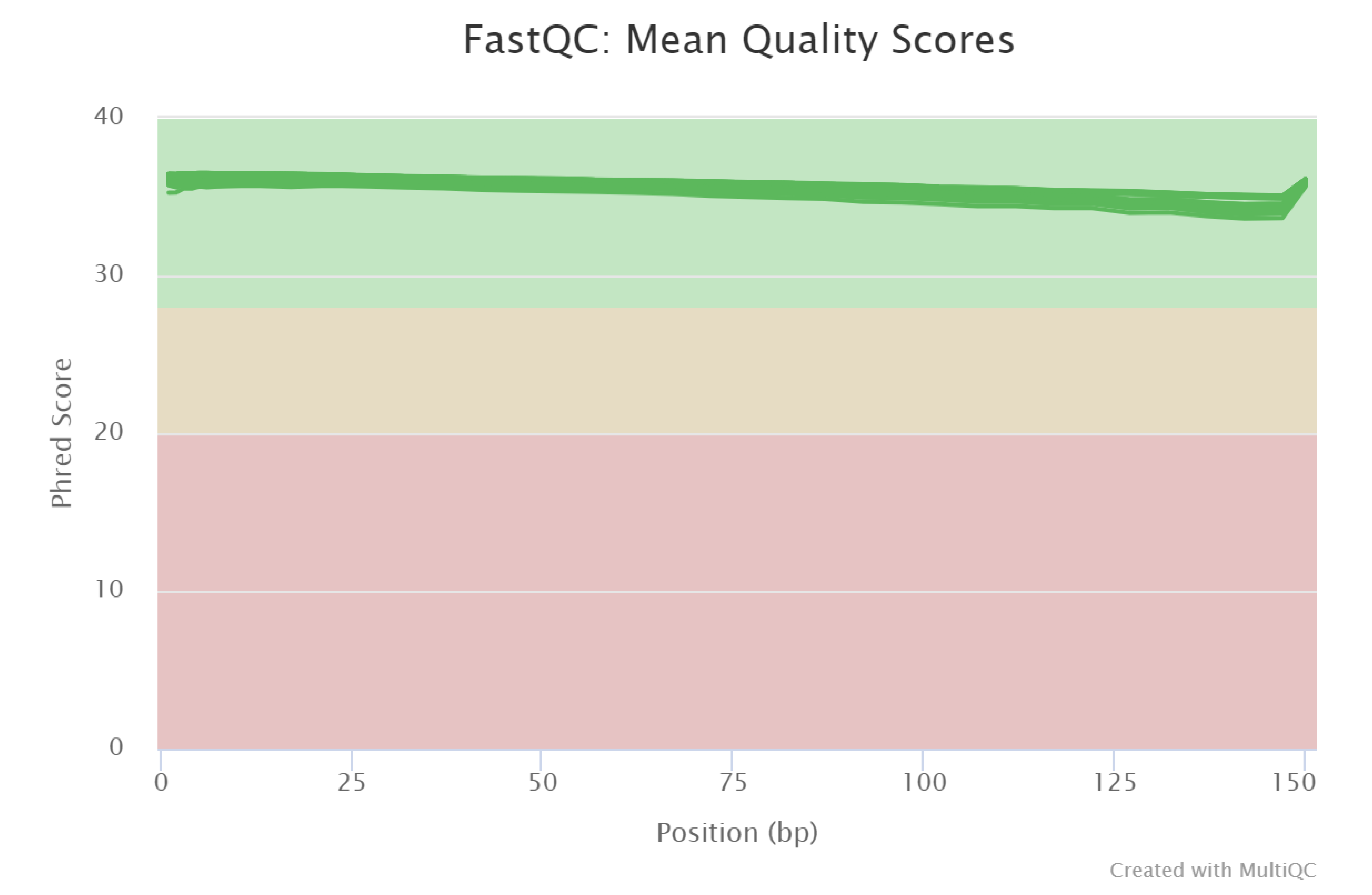

Supplement: Supplementary Figure 1 — All samples are suitable for CNV detection. [file Data_Sheet_1.ZIP › Supplementary Materials/Figure S1.tif]

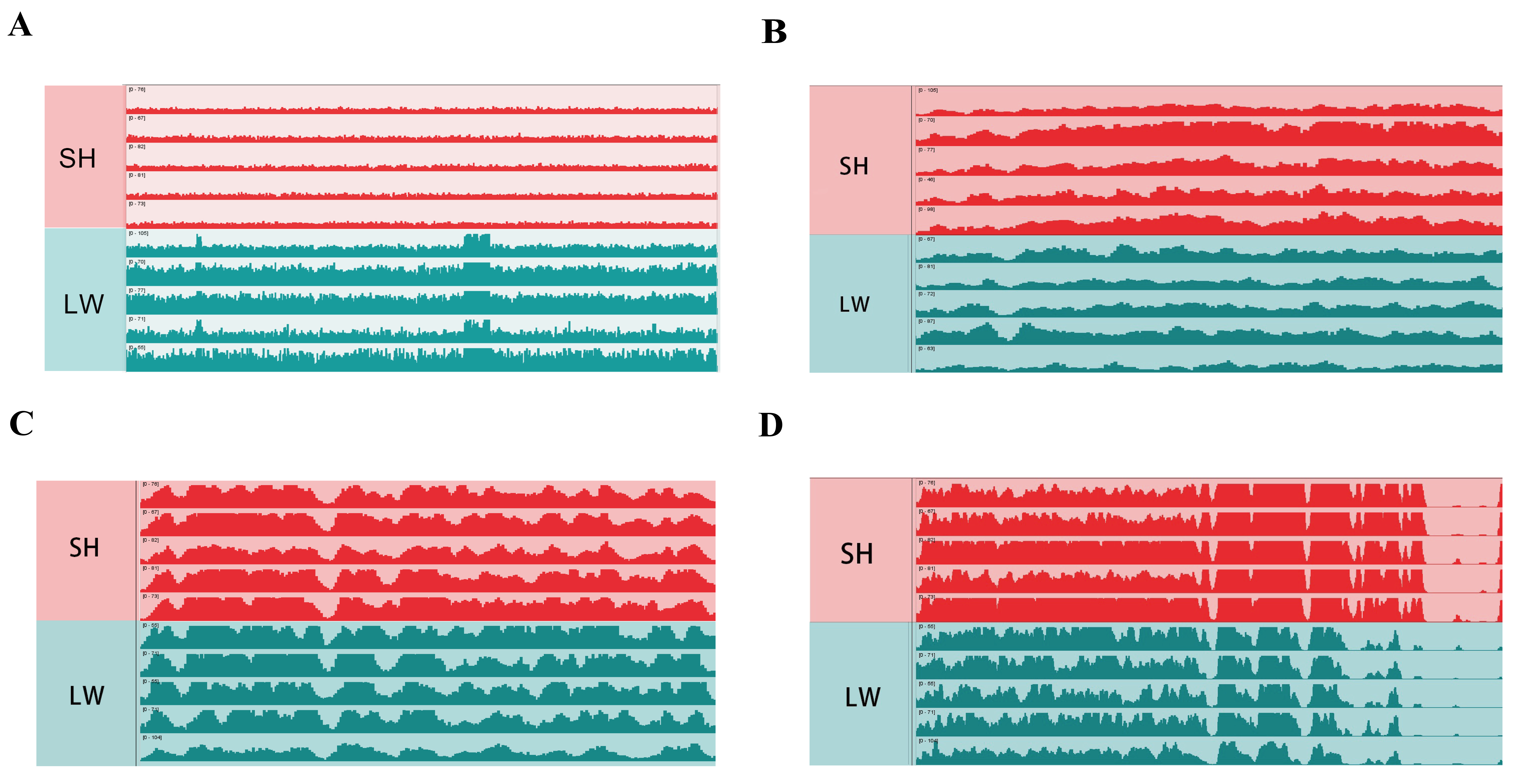

Supplement: Supplementary Figure 1 — All samples are suitable for CNV detection. [file Data_Sheet_1.ZIP › Supplementary Materials/Figure S2.tif]

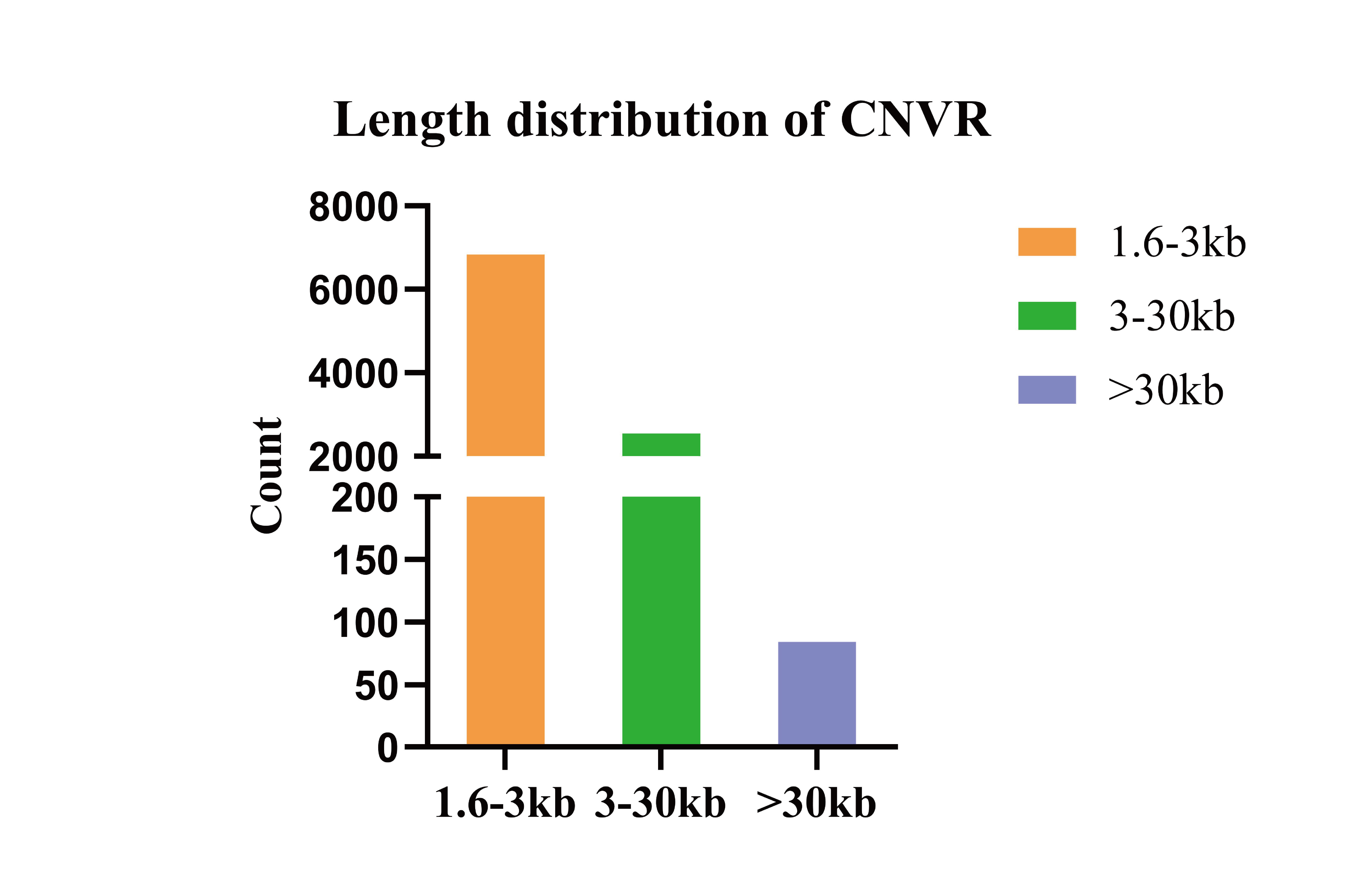

Supplement: Supplementary Figure 1 — All samples are suitable for CNV detection. [file Data_Sheet_1.ZIP › Supplementary Materials/Figure S3.tif]

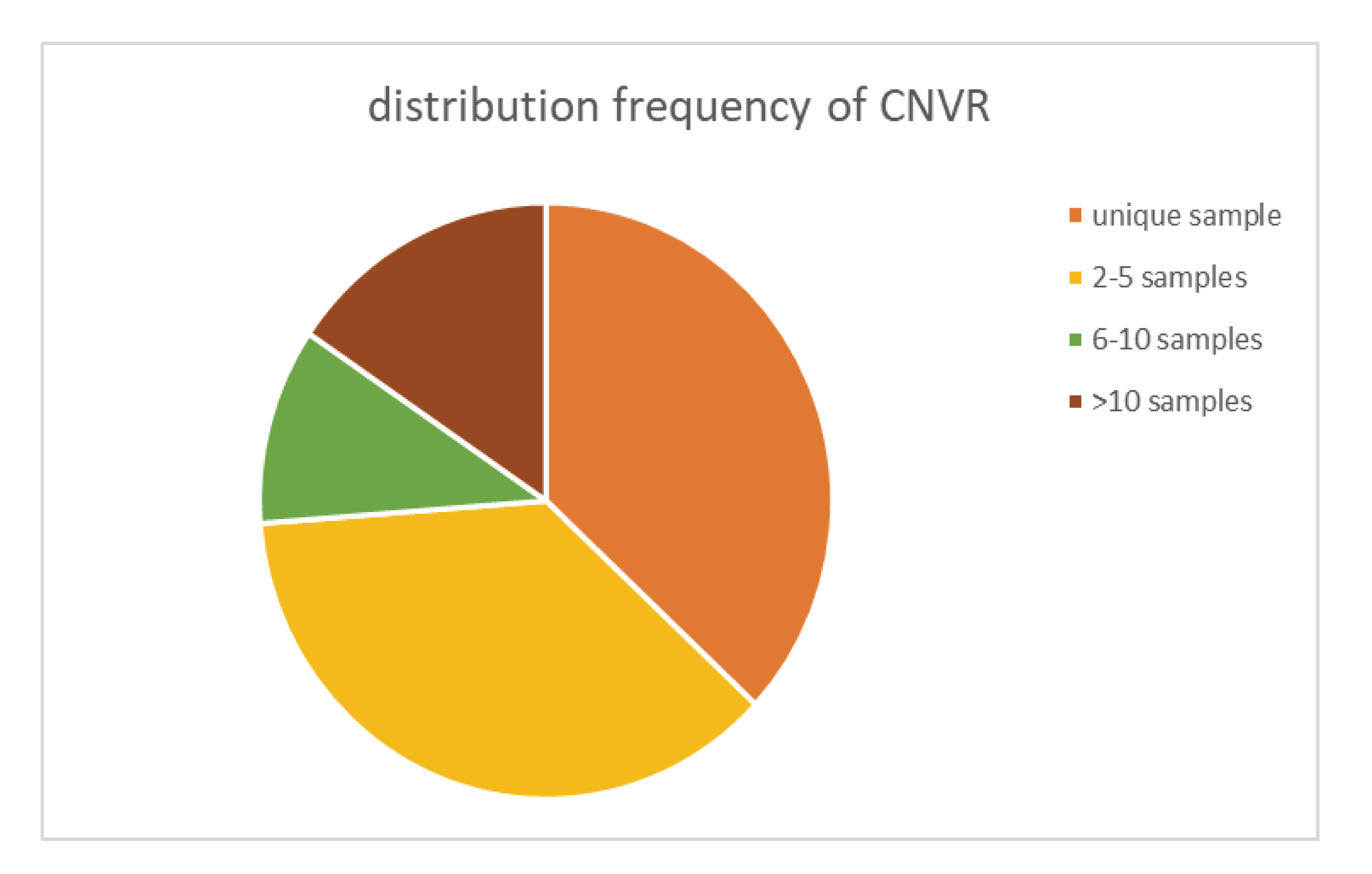

Supplement: Supplementary Figure 1 — All samples are suitable for CNV detection. [file Data_Sheet_1.ZIP › Supplementary Materials/Figure S4.tif]

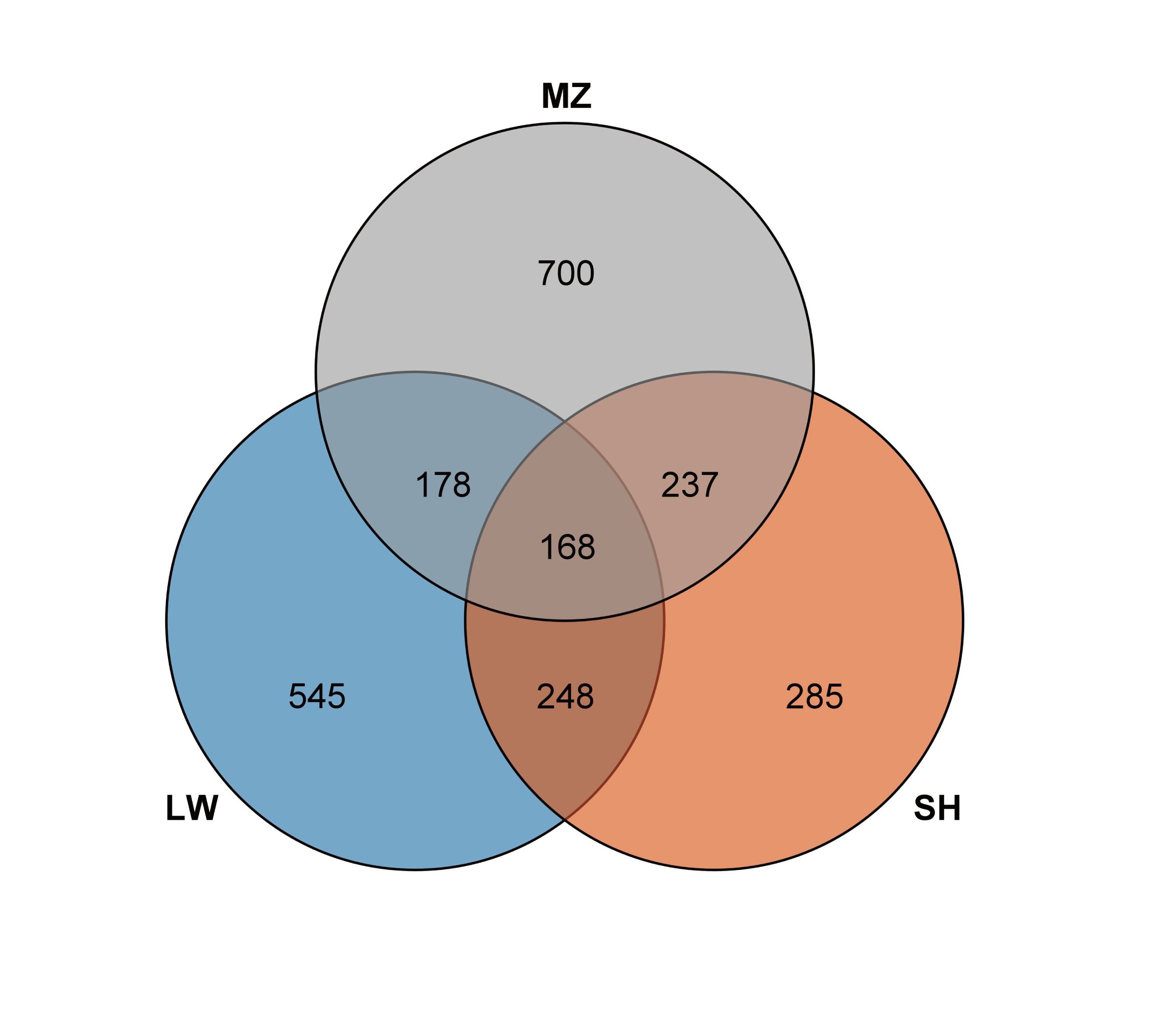

Supplement: Supplementary Figure 1 — All samples are suitable for CNV detection. [file Data_Sheet_1.ZIP › Supplementary Materials/Figure S5.tif]
